# Is monkeypox going to be the next pandemic?

**DOI:** 10.1186/s42269-023-00995-1

**Published:** 2023-02-10

**Authors:** Ridwan Olamilekan Adesola, Joanna Nicole D’Souza, Aisha Bisola Bello, Varaidzo Joyline Mukwekwe

**Affiliations:** 1 Department of Veterinary Medicine, Faculty of Veterinary Medicine, University of Ibadan, Ibadan, Nigeria; 2 Department of Biotechnology, R.V. College of Engineering, Bangalore, 560059 India; 3 Department of Biological Science, Federal Polytechnic Bida, Bida, Nigeria; 4 Parul Institute of Public Health, Faculty of Medicine, Parul University, Waghodia, India

**Keywords:** Monkeypox virus, Non-endemic, COVID-19, Men who have sex with men

## Abstract

**Background and aims:**

In the current global scenario, the monkeypox virus has infected over 3000 individuals from endemic countries like Nigeria, along with non-endemic countries like the UK, Canada, the USA, etc. Based on the current information, it has been observed that monkeypox cases have primarily, though not exclusively, been found among men who have sex with men (MSM) in countries such as the UK. This article discusses the recent outbreak of monkeypox, its causes, and the various approaches to combat monkeypox infections.

**Methods:**

We evaluated the trends of recent outbreaks of monkeypox in different countries and compared them to how the COVID-19 pandemic started.

**Results:**

At present, monkeypox has been reported to spread to over 58 countries via skin-to-skin contact, body fluids, contaminated bed sheets, clothing, or respiratory routes. Smallpox vaccines have been proven to have 85% efficacy against monkeypox. To mitigate this current outbreak, WHO urges people to practice good hygiene and safe sex. The documentation of more cases and further onward spread in the countries in member states are most likely to reoccur, and if not contained, we might experience another global pandemic. Therefore, more research is required to avert this problem.

**Conclusion:**

Monkeypox virus is testing if we have complied with COVID-19 pandemic lessons and elucidates the urgency of research required to understand the monkeypox disease.

## To the Editor,

Monkeypox (MPX) is an endemic, viral zoonotic disease caused by the monkeypox virus (MPVX), with symptoms like smallpox (Alakunle et al. 2020). Despite MPX being a clinically less severe disease than smallpox, it can still lead to many health complications, ranging from skin and eye abnormalities to pneumonia, systemic disorders, and death (Eltvedt et al. 2020). The MPVX is a double-stranded DNA virus (≈ 197 kb). It is a member of the *Orthopoxvirus* genus. It was discovered in 1958 when outbreaks of pox-like disease occurred in a group of monkeys that were used for research purposes. Hence, it got the name 'monkeypox' (Alakunle et al. 2020).

The first human infection was detected in 1970 in a 9-month-old infant boy in Zaire (now the Democratic Republic of the Congo, DRC). Since then, MPX has prevailed in the DRC and spread to other African countries, mainly in Central and West Africa (Alakunle et al. 2020). Outside of Africa, the first cases of MPX were reported in 2003, and the most recent cases were in 2022 (Table 1).

**Table 1:** Countries with past and present monkeypox outbreaks in humans (Alakunle et al. 2020)

Countries	Year of outbreak
Democratic Republic of Congo	1970, 1966–1997, 2001, 2004, 2005, 2007, 2022
Liberia	1970, 2018, 2022
Nigeria	1971, 1978, 2017–2022
Sierra Leone	1970, 2014, 2017
Central African Republic	1983, 2018, 2022
USA	2003, 2021, 2022
UK	2018, 2019, 2021, 2022
Israel	2018, 2022
Cameroon	2018, 2022
Singapore	2019, 2022

Symptoms of the infection include chills, tiredness, headaches, swollen lymph nodes, and fever, which are clinically like the flu. In monkeypox, fever usually begins 1–5 days before rash development. The rashes typically develop on the face and then spread to other body parts, including the soles and the palms. These rashes will then transform into pus-filled blisters, which dry and fall off. The main difference between monkeypox and smallpox is the presence of swollen lymph nodes in monkeypox (Mahase 2022). The severity of monkeypox depends on various factors, such as the age and comorbidities of the patient. Fatality due to monkeypox has been estimated to be as high as 15%, with younger children being the most vulnerable (Eltvedt et al. 2020). Unfortunately, the monkeypox has no treatment available at the moment, but various antiviral drugs were used to treat the disease locally and systemically. The antiviral medicines include Tecovirimat, Cidofovir, and Brincidofovir (Idris and Adesola 2022). This article discusses the recent outbreak of monkeypox, its causes, the various approaches to combat monkeypox infections and answer the question of monkeypox being the next pandemic after Coronavirus disease (COVID-19).

**Fig. 1:**
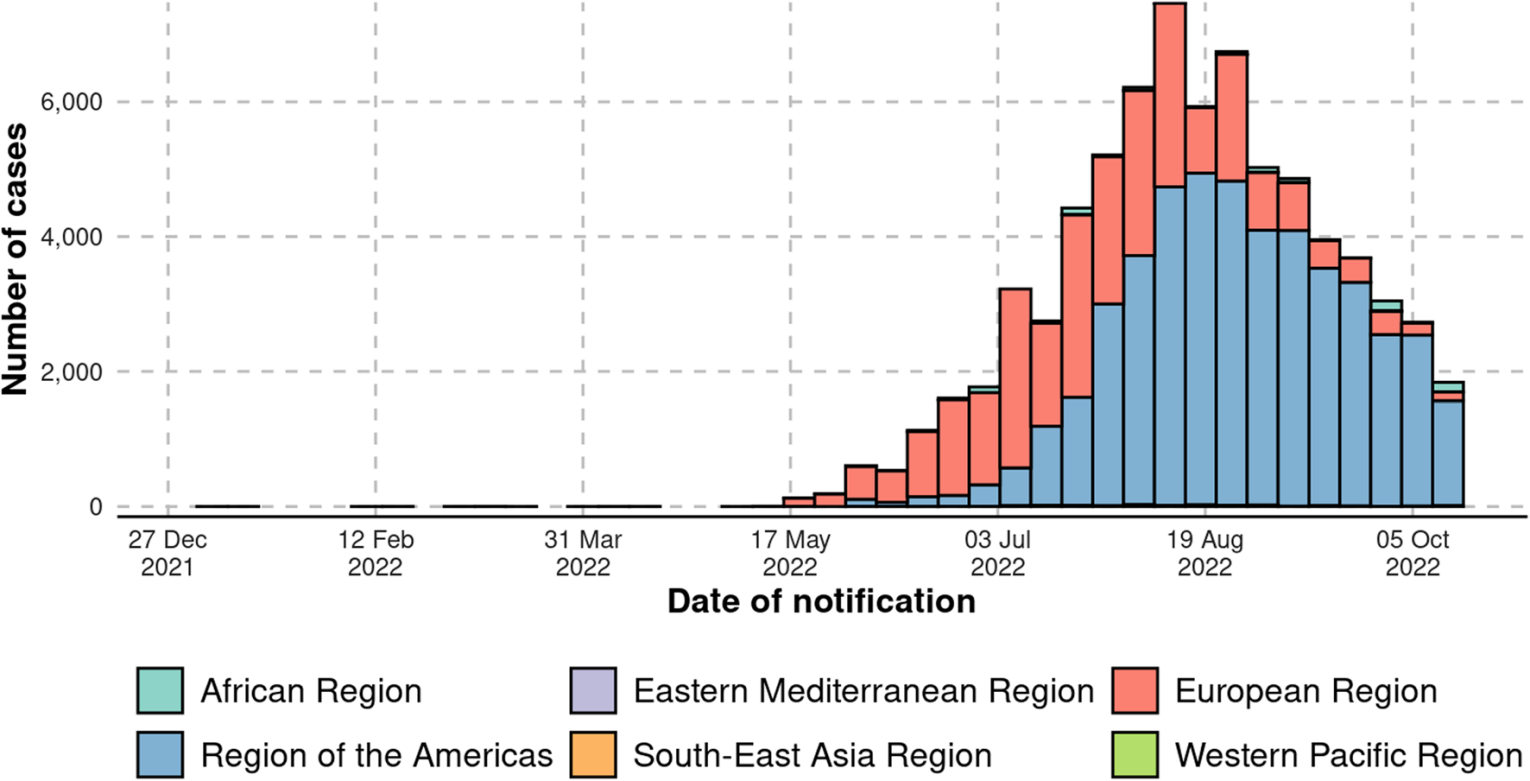
Trend of monkeypox cases from December 2021 to October 2022 (WHO 2022)

On April 29 2022, a British resident, after traveling to Nigeria, showed the primary symptoms related to MPX. On May 4, the resident returned to the UK, becoming the outbreak's index case in the nation. This first case was reported by the UK Health Security Agency on May 7. Eventually, subsequent cases were confirmed in at least 20 countries (Dye and Kraemer 2022). On May 14, two additional cases were established in the UK. The patients resided in the same household. However, none had a history of travel to Africa and had no interaction with the case reported on May 7. Other monkeypox cases have been reported to the WHO regularly from 12 member nations spread over three WHO regions (Fig. 1). On May 21 2022, there were 92 experimentally confirmed cases of monkeypox and 28 suspected cases of monkeypox reported to the WHO from the UK, Canada, the USA, France, Belgium, Germany, Spain, Italy, Portugal, Australia, and Sweden (Zumla et al. 2022). On May 28 2022, there were more than 200 confirmed cases and more than 100 suspected cases of monkeypox that were detected outside of countries where it usually circulates. On June 6, 2022, WHO reported around 780 laboratory-confirmed cases from 27 non-endemic countries. As of June 24 2022, around 3300 cases were reported across 58 countries.

Most affected individuals are young men, many of whom self-identify as men who have sex with men (MSM) and have no recent travel history to endemic areas (Idris and Adesola 2022). Most of these patients had lesions on the genitalia or peri-genital area, implying that transmission mostly happens due to close physical contact during sexual activities. This is the first time that several infections have been recorded in the UK with no known epidemiological links to the disease's endemic areas in West and Central Africa. These are also the first cases of MSM around the globe to be recorded (Mahase 2022; Dye and Kraemer 2022). It is important to note that MPX is not a sexually transmitted disease. This contagious infection can spread through skin-to-skin contact with an infected person with a lesion. It can also spread through body fluids, contaminated bed sheets clothing, or respiratory droplets if a person has a lesion in their mouth (Mahase 2022).

Countless people infected with MPVX have a mild, self-limiting infection course with a shortage of specific treatments. Yet, antivirals developed for smallpox infection may be beneficial in treating MPVX. Smallpox vaccines have been proven to have 85% efficacy against MPVX (CDC 2022). To mitigate this current outbreak, the WHO enjoins people to practice good hygiene and safe sex (Gilchrist 2022), because it is unlikely that mass vaccinations might be required to control the spike in monkeypox cases.

When compared to smallpox, the death rates in monkeypox are much lower. However, it can be predicted that unless actions are implemented to end this ongoing spread, millions of people can die, and many infected individuals can become blind or disabled. This outbreak will not stop without united global action. With the current trend of increasing cases, there is no rationale to wait for the instances to decrease over time. The primary activity to be implemented is to raise awareness of the symptoms and the readily available testing for monkeypox. Any delay in executing these actions can lead to an increase in cases and deaths or even a pandemic.

The documentation of more cases and further onward spread in the countries in member states are most likely to reoccur, and if not contained, we might experience another global pandemic. Therefore, more research is required to avert this problem. An individual suspected of MPX should be thoroughly investigated if confirmed and isolated immediately until the crust falls off. Government should increase awareness among the citizens on the typical lesion of monkeypox, and any suspected cases should be reported to healthcare centers immediately. Additionally, inhabitants and travelers to MPX-endemic countries should avoid physical contact with sick mammals such as monkeys, marsupials, rodents, and non-primates, either alive or dead, that could harbor MPVX and should catchphrase from consumption or handling of bush meat.

## Data Availability

Not applicable.

## Abbreviations

COVID-19: Coronavirus disease
MPVX: Monkeypox virus
MSM: Men who have sex with men
WHO: World Health Organization
DNA: Deoxyribonucleic acid
DRC: Democratic Republic of Congo
MPX: Monkeypox
